# The Commensal *Neisseria musculi* Modulates Host Innate Immunity To Promote Oral Colonization

**DOI:** 10.4049/immunohorizons.1800070

**Published:** 2018-10-31

**Authors:** Daniel A. Powell, Mancheong Ma, Magdalene So, Jeffrey A. Frelinger

**Affiliations:** * Department of Immunobiology, University of Arizona, Tucson, AZ 85724;; † Valley Fever Center for Excellence, University of Arizona, Tucson, AZ 85724;; ‡ BIO5 Institute, University of Arizona, Tucson, AZ 85724

## Abstract

*Neisseria musculi*, isolated from the oral cavity of wild-caught mice, does not colonize most inbred mouse strains. *N. musculi* does weakly (50%) colonize C57BL/6J (B6) mice but readily colonizes CAST/EiJ (CAST) mice. In this study, we examined whether differences in the CAST and B6 host response could elucidate mechanisms governing *N. musculi* colonization. In vivo stimulation of B6 or CAST splenocytes with wild type (WT) *Neisseria* or *Escherichia coli* LPS showed that CAST mice had a blunted inflammatory response, producing significantly lower levels of IL-6 than B6 mice. The use of specific genetic knockouts highlighted a need for an intact innate immune system to prevent colonization. B6–RAG-1^−/−^ mice were colonized at a similar rate as WT B6 mice, whereas B6-MyD88^−/−^ and TLR4^−/−^ mice were readily colonized like CAST (100%) mice. Sequence analysis revealed a unique point mutation in TLR4 in CAST mice. However, crosses to TLR4^−/−^ mice and analysis of recombinant inbred Collaborative Cross mice showed that TLR4 from CAST mice was not sufficient to allow *Neisseria* colonization. In vitro stimulation of B6 bone marrow–derived macrophages or splenocytes with WT *Neisseria* yielded low levels of IL-6 compared with LPS stimulation. Surprisingly, UV-inactivated *Neisseria* induced high levels of IL-6, suggesting suppression of IL-6 production is an active bacterial process. Consistent with a critical role for IL-6 in preventing colonization, mice deficient for the IL-6 receptor were efficiently colonized, indicating host IL-6 production plays a critical role in determining host colonization susceptibility.

## INTRODUCTION

Commensal bacteria are involved in many facets of host processes, including development of the gut immune system and prevention of pathogen colonization. Disruptions of commensal communities are linked to a variety of conditions, including autoimmunity and inflammatory bowel disease (1–7). The factors, both host and bacterial, that allow for colonization remain lightly explored because of the fact that the majority of commensals are not culturable or genetically manipulatable (8–10). As a result, there is sparse literature on the immune response following introduction of a new commensal into adult mice. Indeed, much of the work of the commensal segmented filamentous bacteria relies only on colonization following antibiotic pretreatment (11). Recent work in our laboratory has established a new mouse model for colonization using the recently described mouse commensal *Neisseria musculi* (12, 13). *N. musculi* was isolated from the oral cavity of healthy wild-caught mice.

*N. musculi* is easily grown in vitro and amenable to genetic manipulation. *N. musculi* has conserved many of the host interaction genes that have previously shown to be important for *N. gonorrhea* and *N. meningitis* interaction in in vitro conditions (12,13). A single inoculation of *N. musculi* into the oral cavity of mice produces long term colonization of both the oral cavity and the gastrointestinal tract. Colonization is stable and persistent for at least 1 year, and mice show no signs of disease (12).

Although the *N. musculi* was isolated from wild mice, few strains of laboratory mice were able to be persistently colonized. Two strains tested, CAST and A/J, were readily colonized, with nearly all mice inoculated becoming stably colonized. In contrast, strains NZB, NZO, 129, WSB, and PWK were all highly resistant, with no mice able to persistently harbor *N. musculi*. B6 mice were intermediate in their susceptibility, with ~50% of inoculated mice found to harbor the bacteria for months (12).

Given that we have both susceptible and resistant mouse strains, we were able to examine the differing host responses to *N. musculi* to determine important features underlying *N. musculi* colonization. The partial resistance of B6 mice to *N. musculi* colonization allowed us to use knockout mice on a B6 background to test the role of a variety of genes and pathways in permitting or resisting colonization. To test the general role of innate and adaptive immunity, we inoculated MyD88-deficient mice and found that they are readily colonized, with nearly all mice inoculated becoming persistently colonized. In stark contrast, when we tested the role of adaptive immunity using RAG-deficient mice, we found that these were indistinguishable from B6 mice, with ~50% of inoculated animals becoming persistently colonized. Together, these results suggest a strong role for the innate immune response but relatively little contribution of the adaptive response in the resistance to *N. musculi* colonization (12).

In this manuscript, we extend these previous observations to an examination of additional facets of innate immunity but also show a surprising footprint of the adaptive response in colonized mice. We show a clear impact of the TLR4 signaling pathway. Susceptible strains have a diminished response to both intact *N. musculi* and to the canonical TLR4 ligand LPS. We further show that loss of the downstream effector IL-6 is sufficient to allow colonization. Further, despite the lack of impact on persistence itself, we demonstrate that carriers produce a robust specific IgG Ab response, indicating that the adaptive immune system does recognize the new commensal, but the response is not sufficient to clear the new bacteria.

## MATERIALS AND METHODS

### Mice

See Table I for the source and origin of mice used. All animal protocols were approved by The University of Arizona Institutional Animal Care and Use Committee.

### Colonization of mice

All mice were placed in a specific pathogen free, biosafety level 2 room for 2 wk before inoculation. One week before the inoculation, the oral cavity was swabbed using the BD BBL CultureSwab Plus Transport System (Thermo Fisher Scientific) for indigenous *Neisseria*. Preinoculation swab suspensions in GCB medium base (Becton Dickinson) were plated on GCB agar containing vancomycin (2 μg/ml) and trimethoprim (3 μg/ml), and the plates were incubated for 48 h at 37°C, 5% CO_2_. No *Neisseria* were recovered from any mouse before inoculation (12). On the day of inoculation, AP2365 was swabbed from the agar plate and resuspended in PBS at an OD_600_ of 2.0. Mice were manually restrained, and 50 μl of the bacterial suspension was pipetted into the oral cavity. The oral cavities of the inoculated mice were swabbed weekly or biweekly. Swab suspensions in GCB medium base (Becton Dickinson) were plated on GCB agar containing rifampicin (40 μg/ml), and the plates were incubated for 48 h at 37°C, 5% CO_2_. The colonization frequency of *N. musculi* was calculated from counting the CFU after 48 h.

### Cellular stimulation

Spleens were aseptically removed from 6- to 8-wk-old mice. Spleens were ground over 70-μm cell strainers to produce a single cell suspension. RBCs were lysed with ACK lysis buffer. Remaining cells were resuspended in complete DMEM (10% FBS, 10 mM l-glutamine, 1 mM sodium pyruvate). Bone marrow–derived macrophages (BMDM) transformed by J2 virus were a kind gift from K. Fitzgerald (University of Massachusetts). Cells were seeded into 96-well plates at 2.5 × 10^5^ cells per well in 200 μl of cDMEM. For bacterial stimulations, *N. musculi* AP2365 was grown to mid-log in GCB liquid culture. Bacteria were then pelleted and resuspended in sterile PBS. The suspension was adjusted to give a multiplicity of infection (MOI) of 5 in 50 μl of PBS. Heat-killed cultures were then incubated at 100°C for 30 min. UV-irradiated cultures were exposed to direct UV light for 30 min. Sterility was confirmed by plating. All boiled and UV-inactivated *N. musculi* preparations contained <1 live CFU/50 μl. Ultrapure *Escherichia coli* LPS was purchased from Sigma-Aldrich and derived from *E. coli* O111:B4. *N. musculi* LPS was isolated following the hot phenol method (14,15). Briefly, 500 mg of lyophilized bacterial pellet was solubilized in a 10 mM Tris-Cl buffer (pH 8), with 2% SDS, 4% 2-ME, 20 mg/ml proteinase K, and 2 mM MgCl_2_ at 65°C for 1 h with intermittent vortexing and further digested overnight at 37°C. The samples were precipitated overnight at 20°C with the addition of sodium acetate to a final concentration of 0.1 M and cold ethanol to 75%. LPS was pelleted, and the precipitation was repeated two more times to remove residual SDS and peptides. The samples were then suspended in a 10 mM Tris-Cl buffer (pH 7.4) and digested for 4 h at 37°C with 100 μg/ml DNase and 25 μg/ml RNase. An equal volume of 90% phenol was added, and the sample was incubated at 65°C for 15 min with occasional vortexing. The sample was cooled in ice-water and centrifuged, and the aqueous fraction was collected. The phenol layer was re-extracted with an equal volume of endotoxin-free water. The aqueous layers were pooled and dialyzed in a 1-kDa molecular-mass–cut off dialysis bag with repeated water changes at 4°C to remove phenol over 48 h. The samples were then frozen on dry ice and lyophilized. Dry samples were washed four times with 2:1 (v/v) chloroform-methanol to remove contaminating hydrophilic lipids and re-extracted to remove contaminating lipoproteins (16). Samples were then lyophilized and stored, sealed at room temperature. Cells were stimulated for 18 h at 37°C and 5% CO_2_. At 18 h, supernatantwas harvested and stored at −80°C until analysis. Cell supernatants were analyzed using a BD Mouse Inflammation Kit (Becton Dickinson) per the manufacturer’s instructions.

### Statistical methods

Analysis was evaluated using standard methods indicated in the figure legends, using GraphPad Prism. Log transformations were performed as indicated to create normally distributed data for parametric tests. For the evaluation of the importance of TLR4^CAST^, we preformed standard nonparametric χ^2^ analysis of the relative risk, treating them as in a standard analysis. No associations were significant.

## RESULTS

### N. musculi–*colonized mice produced an Ab response*

**FIGURE 1.**
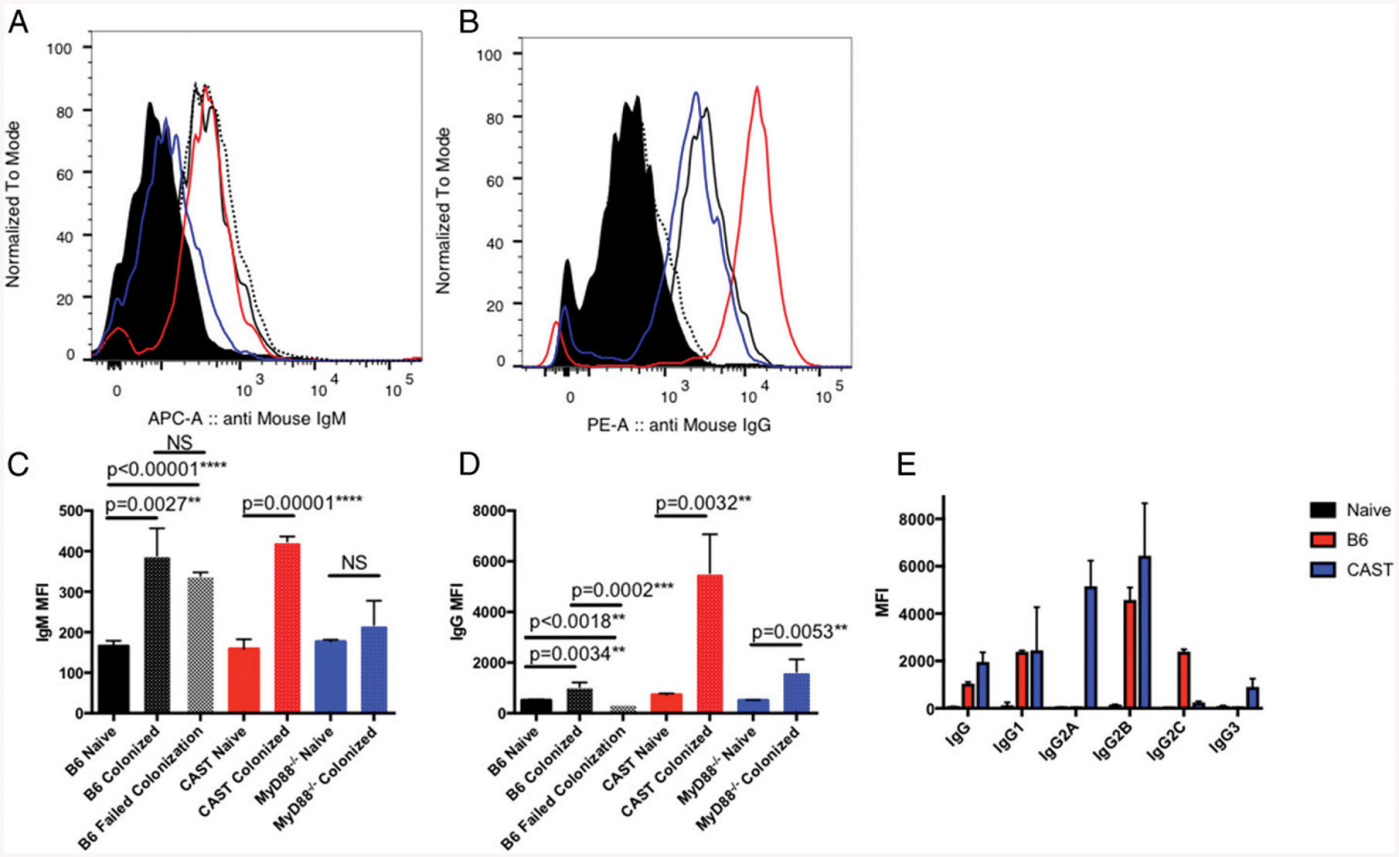
Colonization with *N. musculi* results in Ab production. Representative serum from naive B6 (solid black), colonized B6 (solid black line), B6 mice who failed colonization (dashed black line), colonized MyD88^−/−^ (solid blue line), and colonized CAST (solid red line) was diluted 1:100 and incubated with *N. musculi* AP2365. Secondary Abs against mouse IgM (**A**) and IgG (**B**) were used to determine Ab subclass binding to *N. musculi*. Mean fluorescence intensity (MFI) of IgM (**C**) and IgG (**D**). (*n* = 4–8 mice per group). Plots indicate the mean with SD. Significance was determined using Student *t* test corrected for multiple comparisons. **p < 0.01, ***p < 0.001, ****p < 0.0001. (**E**) Secondary Abs against mouse IgG, IgG1, IgG2A, IgG2B, IgG2C, or IgG3 were used to determine Ab subclass binding to *N. musculi*. Plots indicate the mean with SD of MFI. (*n* = 2–6 mice per group). Data are representative of two independent experiments. There were no significant differences among the strains, except, as expected, by *Igh* alleles.

The major impact of MyD88 deficiency in allowing colonization suggested that *N. musculi* might be undetected by the host immune system. As a simple measure of recognition by the adaptive immune system, we tested colonized mice for *N. musculi*–specific Abs. To detect *N. musculi*–specific Abs, we developed a flow cytometry*–*based binding assay, in which we measured Ab in the serum that was able to bind *N. musculi* cells in vitro and detected binding with a labeled secondary Ab. We originally suspected that colonization of mice in the oral cavity and gastrointestinal tract would not provoke a strong systemic Ab response. Contrary to expectations, colonized CAST mice had *N. musculi*–specific IgM and IgG Abs (Fig. 1A, 1B). Uninoculated CAST and B6 mice had no detectable Ab. Serum Abs were further subtyped to observe IgG subclasses. Both B6 and CAST mice produced IgG1 and IgG2B Abs. Consistent with their *Igh* genes, B6 mice made IgG2C, whereas CAST mice produced *N. musculi*–specific IgG2A Abs. CAST mice produced a small amount of the T cell*–*independent IgG3 subclass; these were not detected in B6 mice (Fig. 1E). This demonstrates that colonization is detected by the adaptive immune response. Because both IgM and IgG are detected, it strongly suggests T cell involvement (17).

### N. musculi–*specific IgG is only produced following successful colonization*

The partial susceptibility of B6 mice to *N. musculi* presented an opportunity to compare Ab responses in inoculated mice that became colonized to those that did not do so. The same inoculum dose was used for all mice. As in CAST mice, colonized B6 mice produced both IgM and T cell–dependent IgG Abs. B6 mice that were not colonized produced IgM Abs (Fig. 1C) but no detectable IgG (Fig. 1D), indicating they had been transiently exposed to *N. musculi* (Fig. 1C). These results show that successful colonization is not an immunologically silent event. The mice that were colonized produce a productive T and B cell response, based on the production of IgG, which requires CD4 T cell involvement. Moreover, *N. musculi* persisted in both the oral cavity and gut of mice in the presence of a B and T cell response. Although we have not determined whether there is robust Ab response in the OC, the presence of an Ab response suggests that a systemic response does not result in clearance of *N. musculi*. Some previous studies have suggested that host Ab response potentiates colonization by bacteria in germ-free mice (18).

### CAST mice respond poorly to MyD88-dependent pathogen associated molecular patterns

**FIGURE 2.**
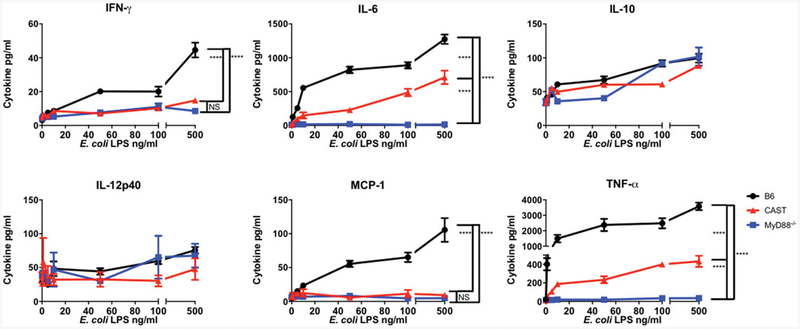
CAST mice have a blunted response to *E. coli* LPS stimulation. Splenocytes from B6 (black circle), CAST (red triangles), and MyD88^−/−^ (blue squares) mice were stimulated for 18 h with increasing concentrations of *E. coli* O111:B5 LPS. Supernatants were analyzed by cytometric bead array. Values are the mean of triplicate wells with SD. Data are representative of three independent experiments. Significance was determined using a Student *t* test on the area under the curve for each mouse strain and corrected for multiple comparisons. ****p < 0.0001.

Both CAST and MyD88-deficient mice are effectively colonized by *N. musculi*. We hypothesized that the lack of MyD88 response in susceptible mice might be the cause of *N. musculi* colonization. We examined the ability of CAST spleen cells to respond to MyD88-dependent PAMPs. Splenocytes from CAST, B6, and MyD88^−/−^ mice were stimulated in vitro for 18 h with increasing concentrations of *E. coli* O111:B4 LPS (TLR4 dependent) or ODN1585 (TLR9 dependent). Supernatants were tested for the following proinflammatory cytokines: IFN-γ, IL-6, IL-10, IL-12p70, MCP-1, and TNF-α. As expected, neither LPS nor ODN1585 induced cytokine production in MyD88^−/−^ splenocytes. IL-12p70 was not detected at any LPS concentration in any of the cultures. As expected, B6 splenocytes responded to LPS by producing IFN-γ, IL-6, MCP-1, and TNF-α in a dose-dependent manner (Fig. 2). CAST splenocytes produced no detectable MCP-1. Although CAST mice did produce some IFN-γ,TNF-α, and IL-6, the amounts were significantly lower than those found in the supernatants of B6-stimulated cells but higher than MyD88^−/−^ cells. In contrast, there was no difference in the response to the TLR9 ligand ODN1585 (Supplemental Fig. 1). These data indicate the TLR4 but not the TLR9 response is blunted in CAST mice compared with B6 mice (Table I).

### CAST mice have a unique single nucleotide polymorphism in the TLR4 coding sequence

CAST mice are an inbred strain derived from the subspecies *Mus musculus castaneus*, and they differ in many loci from the standard inbred mice that are largely derived from *M. musculus musculus* (19, 20). If CAST mice differed from standard inbred strains in their LPS receptor complex (TLR4, MD2, or CD14), it could explain increased CAST mouse colonization susceptibility. Because all of the Collaborative Cross (CC) founder mouse strains tested have had their genomes sequenced, we were able to compare the coding sequences of the relevant genes. We reasoned that for a polymorphism to be relevant, it must be present in CAST but absent in other strains. We found no single nucleotide polymorphisms (SNPs) in MyD88, MD2, or CD14 that are unique to CAST mice (data not shown). However, alignment of their TLR4 loci identified one SNP (C > T) that is unique to CAST. This SNP results in a serine to proline substitution (S312P) in the dimerization domain of CAST TLR4 (Supplemental Fig. 2). This substitution did not result in reduced surface expression of TLR4 as measured by flow cytometry using an anti-TLR4 Ab (data not shown).

We hypothesized that the TLR4 polymorphism might be a determinant of colonization susceptibility. This led us to inoculate TLR4^−/−^ B6 mice with *N. musculi* (Table II). These TLR4^−/−^ mice are as susceptible to *N. musculi* as MyD88^−/−^ B6 mice. C3H/HeJ mice, harboring a spontaneous mutation in TLR4, were also completely susceptible to colonization (Table II), further supporting the argument that TLR4 signaling is involved in susceptibility to *N. musculi* colonization.

### N. musculi *can induce cytokine responses through TLR4*

**FIGURE 3.**
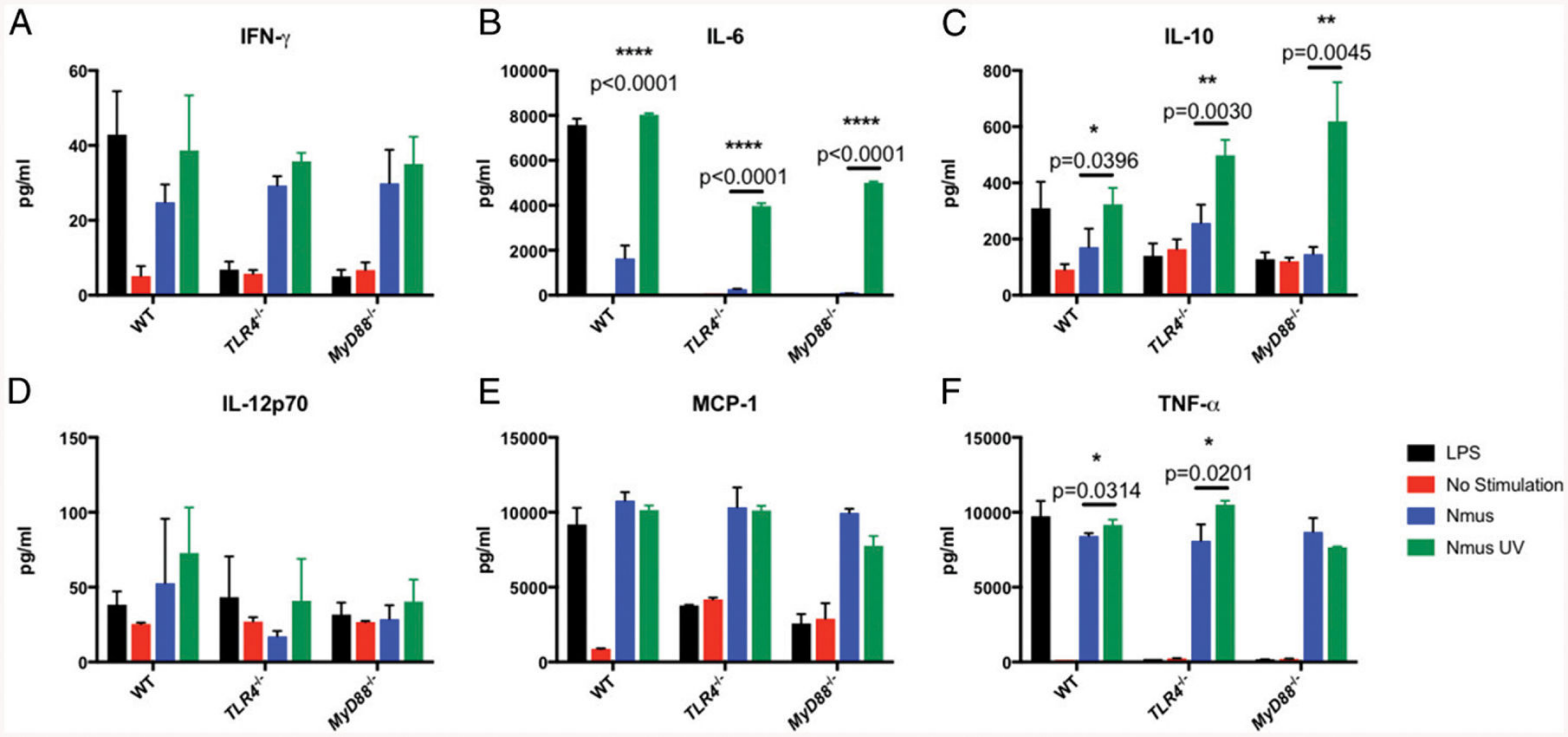
UV-killed *N. musculi* fails to control host IL-6 production. BMDM cell lines derived from WT, TLR4^−/−^, and MyD88^−/−^ mice were stimulated for 18 h with 100 ng/ml of *E. coli* O111:B5 LPS (black bars), live *N. musculi* MOI 5 (blue bars), or UV-killed *N. musculi* MOI 5 (green bars). Supernatants were analyzed by cytometric bead array for IFN-γ (**A**), IL-6 (**B**), IL-10 (**C**), IL-12p70 (**D**), MCP-1 (**E**), and TNF-α (**F**). Values are the average of triplicate wells with SEM. Data are representative of two independent experiments. Significance between live and UV- inactivated *N. musculi* was determined using Student *t* test corrected for multiple comparisons. *p < 0.05, **p < 0.01, ****p < 0.0001.

Because TLR4 and MyD88 signaling deficits correlate with *N. musculi* colonization, we tested the ability of *N. musculi* to stimulate the production of cytokines by immune cells. Because *N. musculi* is a Gram-negative bacterium, we suspected that its LPS would signal through TLR4. We tested the ability of *N. musculi* to signal through TLR4 by using BMDM from B6, TLR4^−/−^, and MyD88^−/−^ mice. We stimulated the cells for 18 h with live *N. musculi*. As a positive control, cells were also stimulated with *E. coli* LPS. The resulting supernatants were tested for proinflammatory cytokines IFN-γ, IL-6, IL-10, IL-12p70, MCP-1, and TNF-α (Fig 3). As expected, MyD88^−/−^ and TLR4^−/−^ cells did not respond to LPS. Of interest, there was similar production of IFN-γ, MCP-1, and TNF-α in response to live *N. musculi* by all three BMDM strains tested, indicating that production of these cytokines was independent of both TLR4 and MyD88.

We then examined the question of whether spleen cells would respond to ligands on the *N. musculi* surface or whether viable bacteria were required. We stimulated spleen cells with UV-inactivated *N. musculi*. Surprisingly, IL-6 was produced when cells were stimulated with UV-killed but not viable *N. musculi*, strongly suggesting live bacteria actively dampen IL-6 production. This finding is consistent with studies showing that bacterial pathogens, including *N. gonorrheae* and *N. meningitidis*, actively perturb host cell signaling during host cell contact to skew infection in their favor (21). The MyD88^−/−^ and TLR4^−/−^ BMDM also produced IL-6 in response to UV-inactivated *N. musculi* but at a lower level than when stimulated by wild type (WT) *N. musculi*. This indicates there are TLR4/MyD88-dependent and -independent signals leading to IL-6 production when stimulated with intact *N. musculi*.

### *CAST cells showed impaired responses to* N. musculi *and* E. coli *LPS*

**FIGURE 4.**
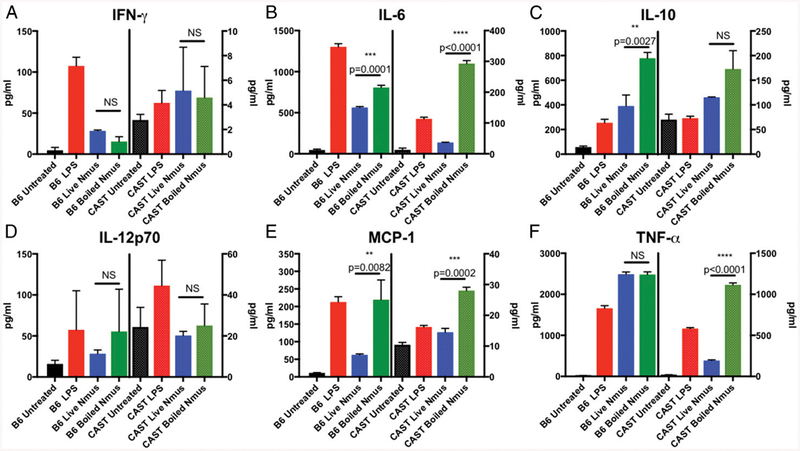
CAST mice have a blunted response to *N. musculi* stimulation. Splenocytes from B6 (left axis) or CAST (right axis) mice were stimulated for 18 h with *E. coli* O111:B4 LPS (red bars), live *N. musculi* MOI 5 (blue bars), or boiled *N. musculi* MOI 5 (green bars). Supernatants were analyzed by cytometric bead array for IFN-γ (**A**), IL-6 (**B**), IL-10 (**C**), IL-12p70 (**D**), MCP-1 (**E**), and TNF-α (**F**). Values are the average of triplicate wells with SEM. Data are representative of two independent experiments. Significance was determined using Student *t* test corrected for multiple comparisons. **p < 0.01, ***p < 0.001, ****p < 0.0001.

Because we showed that *N. musculi* stimulated cytokine production in B6 BMDM, we wanted to determine whether CAST cells were impaired in their ability to respond to *N. musculi* because this would be consistent with their ability to be colonized with *N. musculi*. We stimulated spleen cells from CAST and B6 mice with live *N. musculi*, UV- and heat-killed *N. musculi*, or *E. coli* LPS. The pattern we saw was similar to that seen in Fig. 3. B6 cells responded with IFN-γ, IL-6, IL10, IL-12, MCP-1, and TNF-α. CAST mice were severely impaired in their response to *E. coli* LPS as well as to live and dead *N. musculi* (Fig. 4). Surprisingly, both B6 and CAST mice responded better to killed than live *N. musculi* in their secretion of IL-6, MCP-1, IL10, IL-12, and TNF-α, although not all response differences reached statistical significance. IFN-γ did not show this regulation by live bacteria but was produced more robustly in B6 than CAST. This enhanced response to killed *N. musculi* suggests that there is an active suppression of the inflammatory response by living *N. musculi* in CAST as well as in B6. The response of CAST mice to LPS and *N. musculi* correlates with the ability to colonize, strongly supporting the idea that the defect in TLR4-mediated signaling is a major contributor to the ability to be colonized by *N. musculi*.

### *IL-6 plays a critical role in* N. musculi *colonization*

**FIGURE 5.**
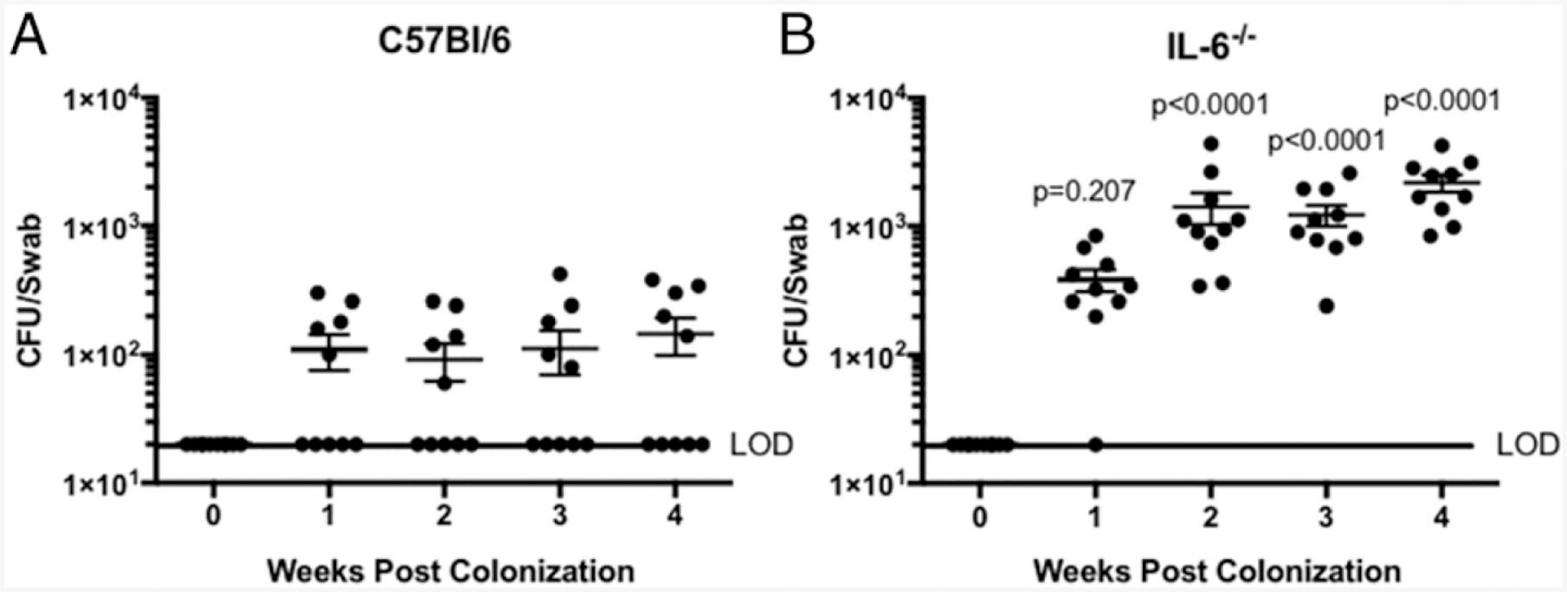
IL-6^−/−^ mice are susceptible to *N. musculi* colonization. CFU of *N. musculi* recovered from the oral cavity of B6 (**A**) or IL-6^**−/−**^ (**B**) mice. Data points indicate individual mice. Data are combined from two individual experiments. Significance was determined using a Student *t* test on log transformed burdens.

The cytokine IL-6 is pivotal in the induction of both innate and adaptive immune responses (22). Because in both CAST and B6 cells the IL-6 response was markedly different between live and killed *N. musculi*, we hypothesized that IL-6 could play an important role in the resistance to colonization. We tested the ability of *N. musculi* to colonize IL-6^−/−^ mice. B6-IL-6^−/−^ mice were very susceptible to colonization (10/10 colonized) and had about a 5-fold higher *N. musculi* burden compared with the B6 parental strain (Fig. 5, Table II). Taken together, these data indicate that inhibition of IL-6 production of by live *N. musculi* is a critical factor in allowing host colonization.

### *TLR4 derived from CAST mice is not the cause of CAST sensitivity to colonization by* N. musculi

The experiments described above strongly suggested the involvement of an innate pathway from TLR4 to MyD88 that resulted in IL-6 production. Indeed, signaling through this pathway mediated by TLR4 was dampened in CAST mice not only when stimulated by whole *N. musculi* and *N. musculi* LPS but by *E. coli* LPS as well. The unique coding sequence polymorphism of TLR4 in CAST mice suggested that an alteration of the TLR4 molecules might result in altered signaling. Consistent with this, the level of surface expression of TLR4 in CAST mice was equivalent to B6 mice, suggesting that the signal transduction but not the expression level was altered at TLR4.

We tested the role of the CAST-derived TLR4 gene in two ways. First, we created F1 mice between TLR4^−/−^ mice and CAST mice. Secondly, we used mice from the CC with CAST TLR4 expressed on a wide variety of different genetic backgrounds (23–25).

**FIGURE 6.**
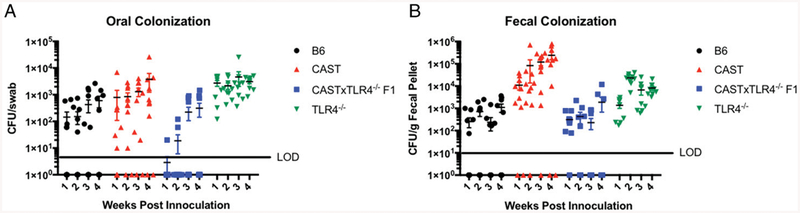
CAST×TLR4^−/−^ mice are resistant to *N. musculi* colonization. CFU of *N. musculi* recovered from the oral cavity (**A**) and fecal pellets (**B**) of B6 (black circles), CAST (red triangles), CAST × TLR4^−/−^ F1 (blue squares), and TLR4^−/−^ (green triangles) mice. Data points indicate individual mice. Data are combined from two individual experiments. Significance was determined using a Student *t* test on log transformed burdens. There was no significant difference between B6 and CAST × TLR4^−/−^ F1 mice.

To initially test the TLR4 CAST association, we crossed CAST mice to B6-TLR4^−/−^ mice. Because the TLR4^−/−^ mice, which are readily colonized, do not express any TLR4, only CAST TLR4 is expressed in these F1 animals. These mice were colonized similarly to B6 mice (4/10), consistent with signaling through TLR4^cast^ being functional and demonstrating the B6 background genes are able to complement the defect in TLR4*–*signaling-defect CAST mice (Fig. 6). It remained possible that there was a gene-dosage effect, and the haploid dose of TLR4^cast^ was not sufficient to allow full colonization.

To examine the possibility that the CAST-derived TLR needed to be homozygous to display the phenotype, we tested mice from the CC for their ability to be colonized by *N. musculi*. The CC is a set of recombinant inbred strains derived from eight different inbred parents. It provides a unique opportunity to test the hypothesis that the CAST mice polymorphism in TLR4 is responsible for susceptibility of CAST mice to *N. musculi* colonization. We selected CC mice whose TLR4 was either derived from CAST (designated TLR4^CAST^ in this study), A/J, or noncolonized parental strains (12). Because the CC mice are all inbred and, hence, homozygous for nearly every allele, we could determine whether homozygous expression of the TLR4 derived from CAST mice was sufficient to allow *N. musculi* colonization.

We tested 18 CC strains, four with TLR4^CAST^ and four with TLR4^A/J^ (Supplemental Table I), for their ability to be colonized with *N. musculi*. We tested all of the available strains with TLR4 genes derived from CAST mice. Overall, 10 of 18 strains were colonized. Five strains were colonized at a frequency of 50%, whereas four strains were colonized at 100%. Three strains of mice that expressed TLR4^CAST^ as well as six that expressed TLR4 from other founder strains were colonized, whereas one, TLR4^CAST^,and seven non-CAST–derived TLR stains were not colonized. Strikingly, strain CC037, which did express TLR4^CAST^, was resistant to colonization. Thus, TLR4 derived from CAST is not sufficient to allow colonization, and other strains that did not express TLR4 from CAST were able to be colonized (Supplemental Table II). Because A/J mice were also colonized, we repeated the analysis excluding the A/J–derived TLR4 mice. In all cases tested, there was no significant association of colonization with either CAST- or A/J–derived TLR4. In Supplemental Table I, we also show the derivation of other relevant TLR4 and MyD88 signaling genes. There was no significant association of any of these alleles with colonization. In principle, this recombinant inbred set could be employed to map the genes for resistance to colonization, but we did not test a sufficient number of the CC strains to make that analysis possible.

Together, these data argue strongly that the susceptibility of CAST and A/J colonization is not due to the polymorphism of their TLR4 genes alone, but, rather, there is an alteration in the pathway. Rather, it suggests that the genetic control is more complex than a simple alteration of the TLR4 protein.

## DISCUSSION

Relatively little effort has been directed at the early events in colonization by commensal bacteria, whereas much has been revealed concerning bacterial pathogens (26). With the discovery of a new mouse commensal, *N. musculi*, we have had the opportunity to examine the early events in the establishment of commensal colonization of adult mice. In this paper, we have found critical pathways in the host response that allow *N. musculi*–successful colonization. B6 mice deficient in either the innate signaling adaptor MyD88 or TLR4 are completely susceptible to colonization with commensal *N. musculi*. Additionally, susceptible CAST mice showed a blunted inflammatory response to TLR4 but not TLR9 ligands compared with B6 mice. This low-level inflammatory response was seen to both live *N. musculi* as well as purified LPS from either *E. coli* or *N. musculi*. CAST’s normal response to the MyD88-dependent TLR9 ligand ODN1585 indicates that the downstream pathway from MyD88 was functional.

The cytokine IL-6 plays an important role in both bacterial and viral infection, in which it can be both pro- and anti-inflammatory (27). Animals deficient in IL-6 are more susceptible to *Streptococcus pneumoniae* (28). Coculture of killed *N. musculi* with myeloid cells induced large quantities of IL-6. This production was independent of the method of *N. musculi* inactivation, indicating that the increased production was not due to the release of internal ligands. The fact that IL-6 was induced only at low levels with live bacteria indicate that the viable *N. musculi* actively controls IL-6 production. Supporting a role for IL-6 in controlling colonization, mice deficient in the IL-6 cytokine were completely colonized. This supports the contention that *N. musculi* regulation of IL-6 production is an important control in its ability to colonize the host. The exact mechanism of this control remains under investigation.

Sequence analysis of CAST mice identified a unique polymorphism in the TLR4 extracellular region outside of the LPS binding pocket and MD2 docking sites. The decreased response to LPS in CAST mice led us to suggest that this polymorphism might be responsible for the decreased inflammatory response and easy colonization by *N. musculi*. We used two genetic approaches to test this hypothesis. (B6-TLR4^−/−^ × CAST) F1 mice were tested for their ability to support *N. musculi* colonization. These mice express only the TLR4^CAST^ but are heterozygous at all loci where CAST and B6 differ. Surprisingly, these mice are not more susceptible to colonization than B6 mice. Thus, expression of TLR4^CAST^ alone (at least in one copy) is not sufficient to allow colonization, and the genetic control must be more complex.

The CC allowed us to interrogate the role of TLR4^CAST^ and colonization with *N. musculi*. Strains were selected based on their TLR4 origin, either CAST or other. These mice were then inoculated with *N. musculi* and followed for colonization. Surprisingly, the TLR4 origin did not influence the ability to be colonized. Mice with TLR4^CAST^ were colonized at similar rates as mice with other TLR4 origins. Additional sequence analysis of other genes in this pathway (MyD88, CD14, MD2) showed no correlation with strain origin and susceptibility. Taken together, these data indicate that, whereas CAST mice have a polymorphism in TLR4 and decreased responses to LPS, the decreased cytokine production leading to *N. musculi* colonization is multifaceted.

## Supplementary Material

1

## Figures and Tables

**TABLE I.** Mice used in this study

Strain	Source
CC002/Unc	University of North Carolina-CC
CC003/Unc	University of North Carolina-CC
CC004/τ/Unc	University of North Carolina-CC
CC006/TauUnc	University of North Carolina-CC
CC007/Unc	University of North Carolina-CC
CC016/GeniUnc	University of North Carolina-CC
CC019/TauUnc	University of North Carolina-CC
CC023/GeniUnc	University of North Carolina-CC
CC037/TauUnc	University of North Carolina-CC
CC038/GeniUnc	University of North Carolina-CC
CC040TauUnc	University of North Carolina-CC
CC042GeniUnc	University of North Carolina-CC
CC045/GeniUnc	University of North Carolina-CC
CC057/Unc	University of North Carolina-CC
CC061/GeniUnc	University of North Carolina-CC
CC065/Unc	University of North Carolina-CC
CC068/TauUnc	University of North Carolina-CC
CC072/TauUnc	University of North Carolina-CC
A/J	The Jackson Laboratory
C57BL/6J	The Jackson Laboratory
129S1/SvImJ	The Jackson Laboratory
NOD/ShiLtJ	The Jackson Laboratory
NZO/HILt/J	The Jackson Laboratory
CAST/EiJ	The Jackson Laboratory
PWK/PhJ	The Jackson Laboratory
WSB/EiJ	The Jackson Laboratory
C3H/HeJ	The Jackson Laboratory
B6.129S2-Il6^tm1Kopf^/J	The Jackson Laboratory
B6.129P2(SJL)-Myd88^tm1.1Defr^/J	The Jackson Laboratory
B6.B10ScN-Tlr4^lps-del^/JthJ	The Jackson Laboratory

**TABLE II.** Colonization of immunodeficient knockout mice

Strain	No. Colonized/Inoculated (%)	*p* Value^a^
C57BL/6J	17/33 (52)	0.003^b^
MyD88^−/−^	21/21 (100)	<0.001^c^
RAG-1^−/−^	3/14 (21)	NS^c^
TLR4^−/−^	24/24 (100)	<0.001^c^
C3H/HeJ	10/10 (100)	0.016^c^
IL-6R^−/−^	10/10 (100)	0.016^c^

a χ^2^ with Yates correction for small numbers and Bonferroni for multiple pairwise comparisons.

b Compared with CAST/EiJ.

c Compared with WT C57BL/6J.

Expanded from Ma et al. (12).

## Abbreviations used in this article:

BMDM: bone marrow–derived macrophage
CC: Collaborative Cross
MOI: multiplicity of infection
SNP: single nucleotide polymorphism
WT: wild type
