# Supplementary material for: The Commensal Neisseria musculi Modulates Host Innate Immunity To Promote Oral Colonization
Source: Immunohorizons. Author manuscript; Available in PMC 2019 Nov 22. (PMC6873461; doi:10.4049/immunohorizons.1800070)
Supplement: 1 [file NIHMS1059542-supplement-1.pdf]

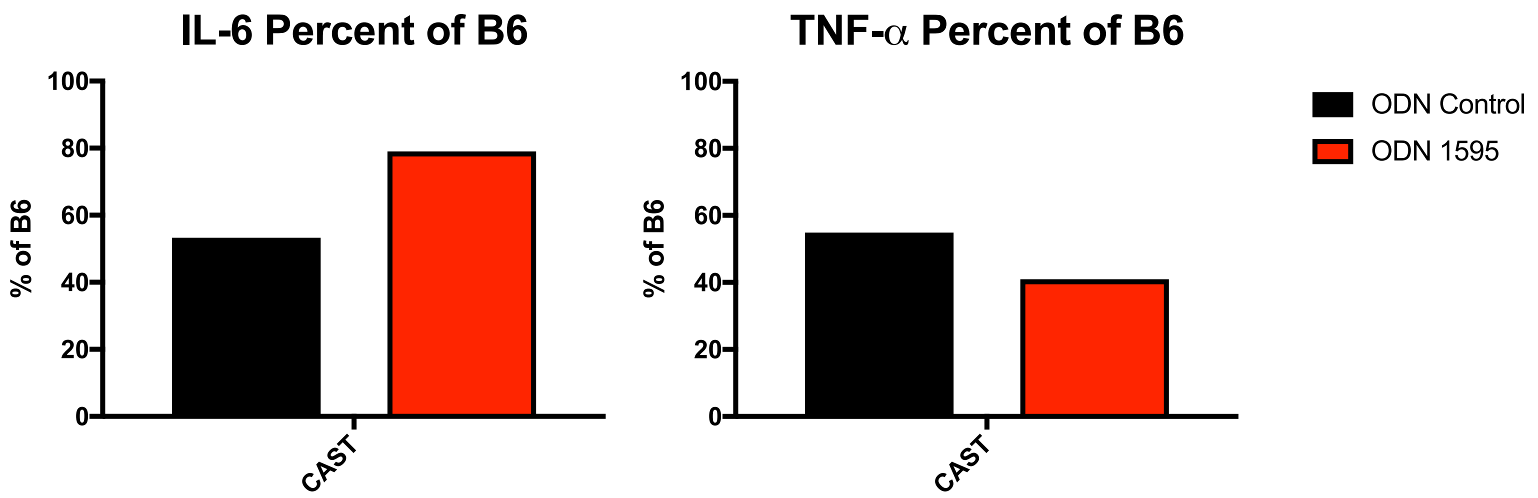

Supplemental Figure 1

CAST mice have similar cytokine production levels compared to B6 after ODN stimulation

Splenocytes from B6 or CAST mice were stimulated for 18 hours with either Control scramble ODN 1585 Control (black bars) or ODN1585 (red bars) at a final concentration of 1 μM. Supernatants were analyzed by cytometric bead array. Values were normalized to B6 spleens stimulated with the ODN 1585 Control.

Alignment of B6 and CAST TLR4 coding sequence. Only the regions with polymorphisms between B6 and CAST shown.
The second plymorphism shown (G>A) is shared by CAST, A/J, PWK, and WSB.

>ENSMUSG00000039005:ENSMUST00000048096 cds:KNOWN_protein_coding

B6: ATACATTCCTGTAAGTTACCTGCATATTT**T**TCCAATCTGACGAACCTAGTACATGTGGAT
CAST: ATACATTCCTGTAAGTTACCTGCATATTT**C**TCCAATCTGACGAACCTAGTACATGTGGAT

B6: CTTTCTTATAACTATATTCAAACTATTACTGTCAACGACTTACAGTTTCTACGTGAAAAT
CAST: CTTTCTTATAACTATATTCAAACTATTACTGTCAACGACTTACAGTTTCTACGTGAAAAT

B6: CCACAAGTCAATCTCTCTTTAGACAT**G**TCTTTGAACCCAATTGACTTCATTCAAGACCAA
CAST: CCACAAGTCAATCTCTCTTTAGACAT**A**TCTTTGAACCCAATTGACTTCATTCAAGACCAA

```
  1 MMPPWLLART LIMALFFSCL TPGSLNPCIE VVPNITYQCM DQKLSKVPDD IPSSTKNIDL
 61 SFNPLKILKS YSFSNFSELQ WLDLSRCEIE TIEDKAWHGL HHLSNLILTG NPIQSFSPGS
121 FSGLTSLENL VAVETKLASL ESFPIGQLIT LKKLNVAHNF IHSCKLPAYF SNLTNLVHVD
181 LSYNYIQTIT VNDLQFLREN PQVNLSLDMS LNPIDFIQDQ AFQGIKLHEL TLRGNFNSSN
241 IMKTCLQNLA GLHVHRLILG EFKDERNLEI FEPSIMEGLC DVTIDEFRLT YTNDFSDDIV
301 KFHCLANVSA MSLAGVSIKY LEDVPKHFKW QSLSIIRCQL KQFPTLDLPF LKSLTLTMNK
361 GSISFKKVAL PSLSYLDLSR NALSFSGCCS YSDLGTNSLR HLDLSFNGAI IMSANFMGLE
421 ELQHLDFQHS TLKRVTEFSA FLSLEKLLYL DISYTNTKID FDGIFLGLTS LNTLKMAGNS
481 FKDNTLSNVF ANTTNLTFLD LSKCQLEQIS WGVFDTLHRL QLLNMSHNNL LFLDSSHYNQ
541 LYSLSTLDCS FNRIETSKGI LQHFPKSLAF FNLTNNSVAC ICEHQKFLQW VKEQKQFLVN
601 VEQMTCATPV EMNTSLVLDF NNSTCYMYKT IISVSVVSVI VVSTVAFLIY HFYFHLILIA
661 GCKKYSRGES IYDAFVIYSS QNEDWVRNEL VKNLEEGVPR FHLCLHYRDF IPGVAIAANI
721 IQEGFHKSRK VIVVVSRHFI QSRWCIFEYE IAQTWQFLSS RSGIIFIVLE KVEKSLLRQQ
781 VELYRLLSRN TYLEWEDNPL GRHIFWRRLK NALLDGKASN PEQTAEEEQE TATWT
```

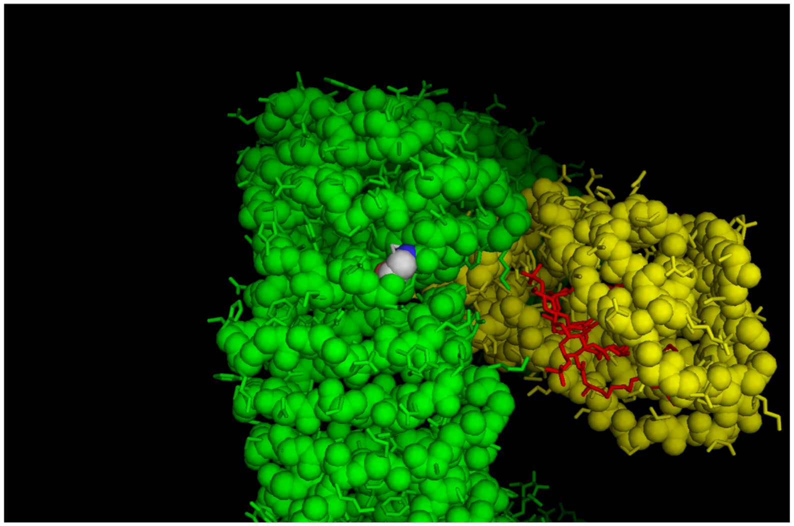

Supplemental Figure 2

Sequence comparison of CAST and B6 TLR4.

DNA and protein alignments of SNPs between CAST and B6 TLR4. Serine 312, indicated in yellow, is the site of the proline substitution in CAST. Space filling model of TLR4 (Green), MD-2 (Yellow), and LPS (Red) the S312P is indicated in white.

CC Number	TLR4 Ori	MD2 (Ly96) Ori	MyD88 Ori	CD14	Colonized	Not Colonized
CC016/GeniUnc	A	“129”	B6	NZO	0	2
CC045/GeniUnc	A	NOD	A	B6	0	2
CC037/TauUnc	CAST	B6	B6	“129”	0	5
CC002/Unc	NZO	B6	CAST	NZO/CAST*	0	2
CC003/Unc	NZO	CAST*	B6	PWK	0	2
CC040TauUnc	NZO	A/CAST*	B6	“129”	0	2
CC042GeniUnc	PWK	NZO	NZO	WSB*	0	1
CC019/TauUnc	WSB	WSB	WSB	A	0	2
CC072/TauUnc	A	NZO	“129”	NZO	1	1
CC038/GeniUnc	CAST	NZO	“129”	NZO	1	1
CC065/Unc	CAST	NZO	“129”	B6	1	1
CC004/Tau/Unc	NOD	“129”	NZO	PWK	1	1
CC007/Unc	NOD	“129”	NZO*	NOD	1	1
CC057/Unc	“129”	NZO	“129”	“129”	2	0
CC023/GeniUnc	A	“129”	PWK	“129”	2	0
CC068/TauUnc	CAST	A	NOD	“129”	2	0
CC006/TauUnc	PWK	*	CAST*	NOD	2	0
CC061/GeniUnc	PWK	PWK	B6 (NOD)	WSB	2	0

Table S1

Effect of TLR^{CAST} on Nmus colonization in CC mice

TLR4 Genotype	Number of Colonized mice	Number of Resistant mice
CAST	4	7
Not CAST	11	16

Table S2
